# Development of an advanced cell therapy product indicated for the treatment of gonarthrosis

**DOI:** 10.1186/1753-6561-9-S9-O9

**Published:** 2015-12-14

**Authors:** Joaquim Vives, Margarita Blanco, Marta Caminal, Maria I Coca, Margarita Codinach, Ruth Coll, Manuel Doral, Mireia Lloret, Irene Oliver-Vila, Isabel Ortega, Laura Reales, Míriam Requena-Montero, Luciano Rodríguez, Sílvia Torrents, Joan García

**Affiliations:** 1 Divisió de Teràpies Avançades/XCELIA, Banc de Sang i Teixits, Passeig Taulat 116, 08005 Barcelona, Spain

## Background

Gonarthrosis is the most common cause of pain and disability in middle-aged and elderly people [1,2]. The lack of long-lasting effective treatments for repairing degenerated articular cartilage has spurred research into novel cell-based therapies aiming at reducing pain, slowing the degeneration of cartilage and, ultimately, reverting the natural history of osteoarthritis (OA) [3]. Herein we report the development of a mesenchymal stromal cell (MSC)-based therapy, from conception up to completion of a Phase I/IIa prospective, open-label, single-dose, single-arm clinical trial.

## Materials and methods

All animal care and experimental procedures adhered to the recommendations of local, national, and European laws and were approved by the appropriate Ethical Committees on Human and Animal Experimentation.

A GMP-compliant bioprocess was designed for the production of the investigational cell-based medicinal product. Characterisation of MSC adhered to the minimal criteria established by the International Society for Cellular Therapy [4].

For the clinical study (EUDRA-CT: 2009-016449-24; http://ClinicalTrials.gov Identifier: NCT01227694), fifteen patients with grade II/ III OA (Kellgren&Lawrence score [5]) and chronic pain were treated intraarticularly with clinical grade MSC and were followed up to 12 months. Primary endpoints were safety and tolerability. Additionally, therapeutic efficacy was measured by the Visual Analogue Scale (VAS) for daily activity and on exertion [6], Health Assessment Questionnaire (HAQ) [7], the SF-36 questionnaire [8], the Western Ontario and McMaster Universities Arthritis (WOMAC) and Lequesne functional indexes[9]. Cartilage integrity was assessed by magnetic resonance imaging (MRI) and T2 relaxation time mapping [10,11].

## Results and discussion

**Figure 1:**
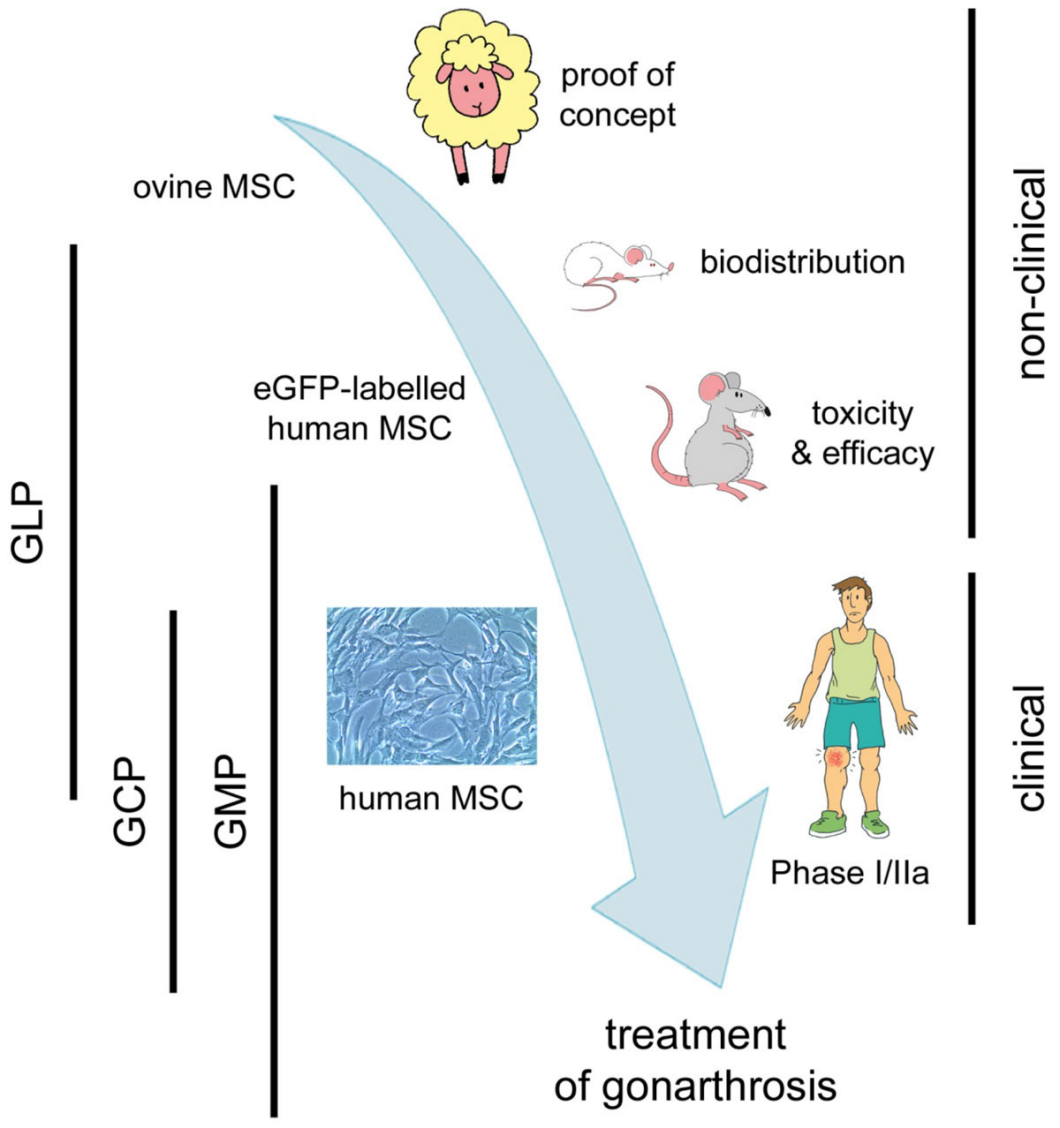
**Schematic representation of the product development package**. eGFP=enhanced Green Fluorescent Protein; GCP=Good Clinical Practice; GLP=Good Laboratory Practice; GMP=Good Manufacturing practice; MSC=Mesenchymal Stromal Cell.

Along the development programme, Good Scientific Practice (GxP) quality standards were implemented gradually in our laboratory (Figure 1)[12].

The preclinical package included a proof-of-principle study in a large animal model of chronic OA and three regulatory preclinical studies in murine animal models (Table 1 Figure 1). The intra-articular injection of autologous MSC was safe, as judged by the lack of local or systemic adverse effects and evidence of regeneration of articular cartilage and meniscus was found in specific macroscopic and histological parameters [13]. Three further regulatory preclinical studies were performed in murine animal models in order to 1) assess subchronic toxicology, 2) analyse the biodistribution of human MSC, and 3) investigate dose:response relationship. Our results highlighted the safety of MSC either administered intra-articularly (up to 6x105MSC/knee in rats) or intravenously (IV, 1.3x107 MSC/kg in mice) and the persistence of IV-infused MSC in liver, kidney and spleen, at 3 months post-administration. No tumours were detected in any of the animals during the observation period. IV transplanted hMSC were principally found in the liver, kidneys and spleen of immunocompromised mice. The lungs, in spite of receiving a considerable number of cells immediately after administration, did not appear to be a welcoming environment adequate for MSC survival, thus confirming other author's observations [14].

**Table 1:** Summary of the product development package for a MSC-based medicinal for the treatment of gonarthrosis.

GLP	GMP	GCP	Study description	Experimental system	Dose; Route of administration
			Assessment of long-term effects of autologous MSC treatment in an ovine chronic model of OA [13]	*Ovis aries* (Ripollesa breed, ♀); surgically-induced OA	11x10^6 ^oMSC; intraarticular
X	X		Biodistribution of hMSC in an immunodeficient mouse model	*Mus musculus* (NRG, ♀ and ♂)	4x10^5 ^hMSC; tail-vein injection
	X		Single-dose toxicology study of intraarticularly administered hMSC in athymic rats	*Rattus norvegicus* (NIH nude, ♂)	Up to 6x10^5 ^hMSC; intraarticular
X	X		Dose-response study after intraarticular injection of hMSC in a rat model of OA	*Rattus norvegicus* (NIH nude, ♂); MIA-induced OA	Up to 8x10^4 ^hMSC; intraarticular
X	X		Analysis of protooncogen expression levels, hTERT activity, senescense, G-banding karyotype, and CGH arrays on clinical grade hMSC	*In vitro *assays	N/A
	X	X	Adult Stem Cell Therapy for Repairing Articular Cartilage in Gonarthrosis (EUDRA-CT: 2009-016449-24; ClinicalTrials.gov Identifier: NCT01227694)	Human(♀ and ♂); grade II/III knee OA	40.9x10^6 ^hMSC; intraarticular

GxP compliance of non-clinical and clinical studies, and for the production of batches of MSC are also indicated. GCP=Good Clinical Practice; GLP=Good Laboratory Practice; GMP=Good Manufacturing practice; MIA=monoiodoacetate; MSC=Mesenchymal Stromal Cell; OA=osteoarthritis; N/A=Not Applicable; NIH=National Institutes of Health; NRG=NOD.Cg-Rag1^tm1Mom ^Il2rg^tm1Wjl^/SzJ.

Previous data in large translational animal models [15], and investigations on the role of MSC in cartilage [16,17] and bone [18] regeneration, provided further support with respect to the safety and regenerative qualities of MSC.

For the clinical testing in humans, the drug product consisted in 40.9x106 ± 0.4x106 viable MSC in 10.0 ± 0.3 mL of saline solution. The phenotypic characteristics of the human MSC used in the study were 99.7% ± 0.2% CD45-CD105+, 99.0% ± 0.6% CD31-CD73+, 99.9 ± 0.3% CD90 and 15.5% ± 14.8 HLA-DR+. The combination of differentiation assays, growth profiles, morphology assessment and cytometric phenotype confirmed the MSC nature of the cells used in our studies. MSC suspensions tested negative for bacteria, mycoplasma and endotoxin before infusion into humans. The analgesic effect of the intra-articular infusion of MSC was remarkable, with all patients showing some degree of improvement in daily life physical activity and on exertion at month 12. Indeed all WOMAC, Lequesne and VAS indexes decreased in a similar manner over time, the most significant changes being observed at 6 and 12 months after treatment. A significant improvement was observed at 12 months in the vitality scale and at 3 months in the global health scale. HAQ decreased significantly from 0.38 at the basal visit to 0.2 at 12 months (p<0.05), thus indicating a global improvement of the perceived health status.

With respect to cartilage integrity, T2 values decreased significantly over time in all patients. These results may be indicative of regeneration of the articular cartilage in all patients at 1-year post-treatment. The fact that no pathological values were observed at 12 months in prior healthy areas highlighted the preventive effect of MSC on further degeneration.

## Conclusions

We successfully designed and executed a reproducible GMP-compliant bioprocess for the manufacture of cell-based therapeutics. The clinical procedure involved a minimally invasive intervention, which was feasible and safe, resulting in pain relief and preventing further degeneration of articular cartilage.

## Acknowledgements

The authors would like to express their sincere gratitude to F. Gòdia, J.J. Cairó, and L. Orozcoand his team for their support in this project. This work was supported by grants ''Ministerio de Economía y Competitividad'' (IPT-300000-2010-0017), ''Ministerio de Ciencia e Innovación'' (PSE-010000-2007-4//PSE-010000-2008-4, BIO2008-01985), Spanish Cell Therapy Network(TerCel, RD12/0019/0015) and by the European Regional Development Fund, within the National Plan for Scientific Research, Development and Innovation 2008-2011.
